# Recognition Method of Knob Gear in Substation Based on YOLOv4 and Darknet53-DUC-DSNT

**DOI:** 10.3390/s22134722

**Published:** 2022-06-22

**Authors:** Ronglin Qin, Zexi Hua, Ziwei Sun, Rujiang He

**Affiliations:** 1 School of Computing and Artificial Intelligence, Southwest Jiaotong University, Chengdu 610032, China; qrl.swjtu.edu.cn@my.swjtu.edu.cn; 2 School of Information Science and Technology, Southwest Jiaotong University, Chengdu 610032, China; sun_zi_wei@my.swjtu.edu.cn (Z.S.); hrj@my.swjtu.edu.cn (R.H.); 3 Qianghua Times (Chengdu) Technology Co., Ltd., Chengdu 610095, China

**Keywords:** knob gear recognition, target detection, key point detection, image classification, YOLOv4, Darknet53

## Abstract

When unattended substations are popular, the knob is a vital monitoring object for unattended substations. However, in the actual scene of the substation, the recognition method of a knob gear has low accuracy. The main reasons are as follows. Firstly, the SNR of knob images is low due to the influence of lighting conditions, which are challenging to extract image features. Secondly, the image deviates from the front view affected by the shooting angle; that knob has a certain deformation, which causes the feature judgment to be disturbed. Finally, the feature distribution of each kind of knob is inconsistent, which interferes with image extraction features and leads to weak spatial generalization ability. For the above problems, we propose a three-stage knob gear recognition method based on YOLOv4 and Darknet53-DUC-DSNT models for the first time and apply key point detection of deep learning to knob gear recognition for the first time. Firstly, YOLOv4 is used as the knob area detector to find knobs from a picture of a cabinet panel. Then, Darknet53, which can extract features, is used as the backbone network for keypoint detection of knobs, combined with DUC structure to recover detailed information and DSNT structure to enhance feature extraction and improve spatial generalization ability. Finally, we obtained the knob gear by calculating the angle between the line of the rotating center point and the pointing point and horizontal direction. The experimental results show that this method effectively solves the above problems and improves the performance of knob gear detection.

## 1. Introduction

With the power system’s rapid development, the substations’ scale has become larger [1]. The traditional method of employing personnel to inspect is no longer suitable for managing much equipment in substations [2,3]. Hence, unattended substations develop rapidly [4]. Among them, automatic knob gear detection is one of the crucial tasks to inspect large-scale equipment in substations automatically. It can automatically obtain the operational status of large-scale and complex equipment, thereby replacing manual inspection and transcription operations. Although there are some methods of knob gear detection, it is still challenging due to the complexity of the actual scene of unattended substations. Therefore, we need to improve the accuracy of knob gear recognition as much as possible based on satisfying the real-time monitoring of substation knobs.

At present, there are few studies on knob gear recognition. By analyzing and summarizing the found articles on knob gear recognition, we generally divided the knob gear recognition methods into two types. The first is an image processing method based on OpenCV [5,6,7,8], which binarizes the image, removes background interference, and obtains knob contour information through Hough detection. Although the methods based on OpenCV can identify the gears of knobs, their performance in complex environments is relatively poor, and they are not universal. Different shapes of knobs require different parameters. Although it is the same type of knob, it will also have a large error due to environmental factors such as lighting [9]. The second method is influenced by the excellent performance of a convolutional neural network (CNN) [10] on image classification [11], and a general object detection algorithm based on CNN is proposed [9,12,13,14,15,16,17,18,19,20]. This kind of algorithm [21,22] has two stages. The first stage is to detect the target of the knob, and the second stage is to classify the knob. This general detector improves gears’ detection accuracy and meets real-time detection requirements but ignores the fine-grained features of images. When the image is in an oblique view, the model will make mistakes in judging the features.

Based on the above article, the images collected in the actual scene were analyzed, and we found the following three problems.

**Figure 1:**
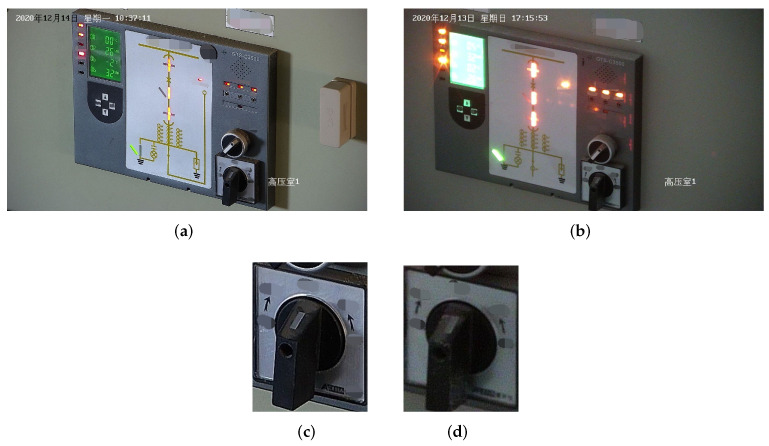
Images under different lighting conditions. (**a**) Image with good lighting conditions. The upper left corner of the image shows the time of acquisition: Monday, 14 December 2020. The lower right corner of the picture shows the location of the acquisition: the first hyperbaric chamber. (**b**) Image with poor lighting conditions. The upper left corner of the image shows the time of collection: Sunday, 13 December 2020. The lower right corner of the picture shows the location of the acquisition: the first hyperbaric chamber. (**c**) Knob image cropped from (**a**) with high SNR. (**d**) Knob image cropped from (**b**) with low SNR.

**Figure 2:**
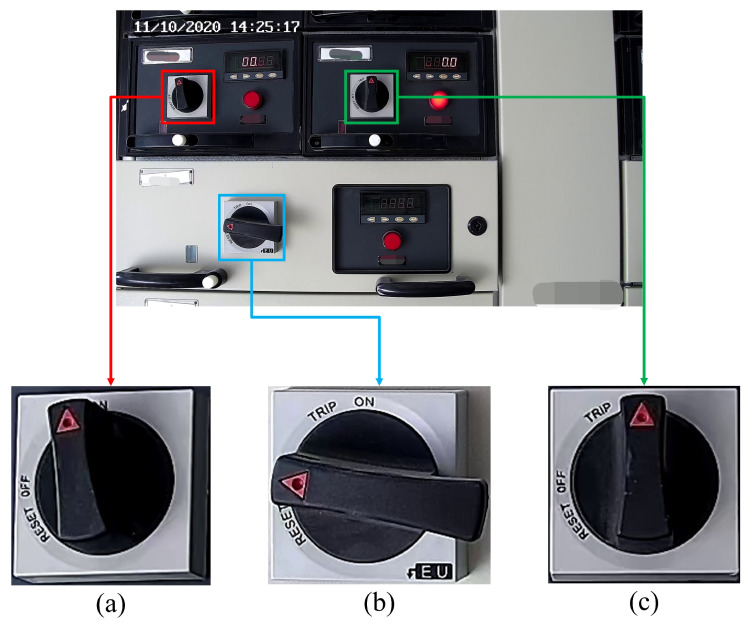
Collected original image at the substation site. (**a**) The oblique view where the actual direction is up and is misjudged as the upper left direction. (**b**) The oblique view where the actual direction is left and is misjudged as the upper left direction. (**c**) Front view.

**Figure 3:**
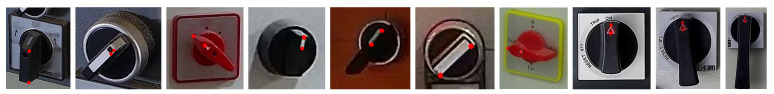
Ten different types of knobs with different feature distributions of key points.

1. Firstly, the quality of images collected in the actual scene of the substation has different levels. The main reason is that the poor lighting conditions cause some images to have a low signal–noise ratio (SNR), which affects the image extraction features. As shown in Figure 1c,d the image in (c) is evident due to the excellent lighting conditions, and the image features are easier to extract. In contrast, it is already challenging to see the features of the knob in (d) due to poor lighting conditions.
2. Secondly, many oblique views are in the images collected from the substation. The knob will be deformed to a certain extent, resulting in inaccurate feature extraction and weak spatial generalization ability. Figure 2 shows the original image collected from the substation. There are three knobs in the same image, of which (a) and (b) are oblique views, and (c) is a front view. Experiments show that if we use the two-stage gear detection model in the article [21] which first locates the knob area and then directly classifies the knob, the model will have a high probability of misidentifying (a) and (b) as the upper left direction, the direction of (a) is up, and the direction of (b) is to the left.
3. Thirdly, due to the variety of knobs, the spatial distribution of key points of each kind of knob is different, as shown in Figure 3. This results in a relatively weak spatial generalization ability of the trained model. Even if the regression coordinate error is only a few pixels, it will still cause misjudgment of the knob gear.

For the first image quality problem, we use the DUC (dense upsampling convolution) [23,24] structure to generate pixel-level predictions, which can compensate for the loss in the length and width dimensions through the channel dimension. PixleShuffle [25] in DUC can convert a low-resolution input image into a high-resolution image by upsampling, thereby compensating for the problem of fine-grained feature loss due to low image SNR. The second and third problems are essentially the same problem. They are both problems of weak spatial generalization ability caused by interference in feature extraction. It is necessary to strengthen feature extraction and improve spatial generalization ability. We further introduce the DSNT (differentiable spatial to numerical transform) [26,27,28,29] module in which the coordinate regression of the knob key points is enhanced through the normalized Gaussian heatmap so that the error of the regression coordinates of the key points is controlled within 1 pixel as much as possible, and the spatial generalization ability of the model is enhanced. Both DUC and DSNT are essential in our model.

Our method achieves better performance than the current knob gear detection methods. These contributions can be summarized as follows.

1. We propose a three-stage knob gear detection method YOLOv4 and Darknet53-DUC-DSNT model for the first time and apply key point detection of deep learning to the knob gear detection for the first time, and the results are more accurate than the two-stage detection.
2. We combine the DUC structure to solve the loss of detail information due to low image SNR.
3. We use the DSNT structure to solve the problem of the key point coordinate regression deviation and weak spatial generalization ability caused by image squint and different feature distributions of key points.

The remainder of this article is organized as follows. Section 2 is to investigate the related work on the knob gear detection methods. Section 3 explicitly describes the three-stage knob gear recognition model proposed by us. Section 4 presents model experiments and results. Section 5 is the conclusion of this article.

## 2. Related Work

At present, there are few studies on knob gear recognition. We summarized the found articles on knob gear recognition and can generally divide the methods into two types. The first is the image processing methods based on OpenCV [5,6,7], such as the method proposed by Rong Cai et al. [5] that divided the knob gear recognition into two parts: rough positioning and precise positioning. They used template matching for the rough positioning [30] method to locate the approximate position of the knob switch in the panorama. After completing the rough positioning, they carried out the precise positioning of the knob area. Precise positioning uses mean-shift filtering [31] to connect pixels of similar patterns in the image. It then uses the flooding method [8] to divide the connected pixels into different regions to separate the target and the background and generate a binary image. Finally, it finds the contour that meets the conditions in which pixels of the shape are the set of rotating center pixels. Rong Cai’s method can only find a collection of key points rather than being accurate to a specific key point, which will cause a significant error in the results. Yanming Wu et al. [6] proposed first to classify different buttons by color and then use the Hough circle algorithm [32] to detect the outline of a circular knob that obtains coordinates of the center of the circle for knob positioning. Then it pastes a blue rectangular bar on the knob, and binarizes the image to obtain the outline of the rectangle and coordinates of the vertices of the rectangle. Yanming Wu’s method is only suitable for detecting circular knobs, and it is necessary to manually attach a rectangular bar to the knob to obtain the vertex coordinates of the rectangle. This method is complicated and not universal. Yulun Wu [7] proposed to extract the knob indicator line of the image through three steps: gray processing, binarization, and erosion. At the same time, they removed the interfering information of the image, and finally used the Hough line transform [33] to identify where the knob indicator line was located and obtained the angle information to determine the knob gear. The image processing methods based on OpenCV have a common disadvantage: the algorithm is not universal and is often only applicable to a specific type of knob that uses different parameters for different knobs. Even with the same knob, the model parameters must be constantly adjusted if the background factors such as lighting conditions are not the same.

The second method is based on deep learning. Mengan Shi et al. [21] proposed first using the YOLO-tiny-RFB model for knob target detection, then the method based on MobileNet [34] is used for knob area to classify various states of knob gear accurately. This method improves the accuracy of knob recognition and has good generalization. Still, the article states that the model ignores the fine-grained features of the image, and the shooting angle of vision also significantly impacts the model’s judgment. The model [21] cannot accurately determine the correct direction of the knob when it deviates from the frontal viewing angle, or the knob has deformation. Zhiling Zhu et al. [22] integrated the recognition algorithm of OpenCV and deep learning. Firstly, they used the improved Canny algorithm [35] to extract the actual edge and combined the perspective transformation to correct the instrument panel image. Secondly, they used the enhanced YOLOv4 algorithm to segment the knob area accurately. Finally, they extracted the pixel contour of the knob groove, the PCA algorithm fit the contour rectangle, and the pose was measured. This method corrects the panel image and solves the problem in [21] that the model cannot accurately judge the gear of the knob when the knob is deformed due to the deviation of the shooting angle. However, the article [22] still uses the OpenCV method to measure the pose, which leads to the fact that the gear detection is still affected by factors such as the type of knob and illumination, reducing the model’s generality.

Aiming at the problems of low image SNR caused by the influence of illumination in the actual scene of the substation and weak spatial generalization caused by image strabismus and different distribution of knob features, we innovatively combine the YOLOv4 [15,16,17] target detection algorithm with the Darknet53 [36,37,38] feature extraction network, the DUC module, and the DSNT module to form a knob gear detection network. We conduct experiments on the knob image data collected in the substation, comparing it with other researchers’ knob gear detection methods. Experiments show that our algorithm can effectively improve the detection accuracy of fine-grained features of images and has better performance than the current knob gear detection methods.

## 3. Knob Gear Recognition Model

**Figure 4:**
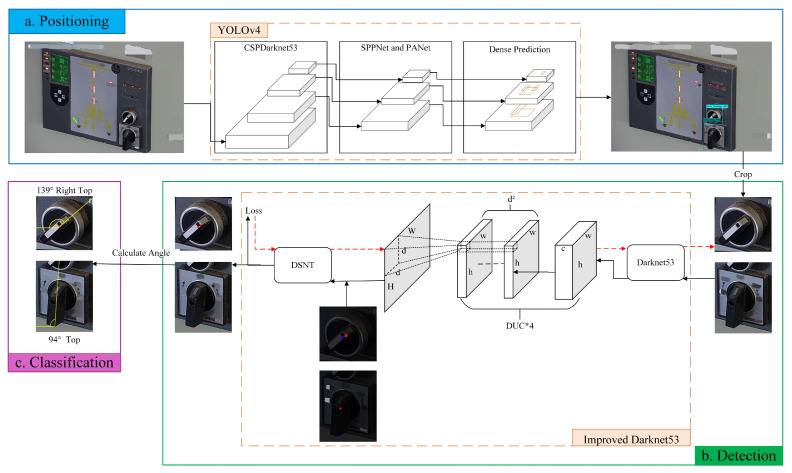
The overall framework of the knob gear recognition model. Panel (**a**) is the knob positioning module; (**b**) is the detection module of the key point of the knob; (**c**) is the knob gear classification module.

Figure 4 shows the overall framework of the knob gear recognition model. First of all, since the original image collected at the substation is a complete cabinet panel image that includes components such as miniature lights, knobs, digital meters, and so on, it is impossible to identify the gear of knobs on the original image directly. Hence, the knob area must be positioned and cropped out by YOLOv4 first. Then, feature extraction and coordinate regression is performed on the cropped knob data through the key point detection algorithm and it predicts the coordinates of two key points of the knob. Finally, after predicting the coordinates of the two key points, the angle between the straight line of the two key points and the horizontal direction is calculated, and it is determined which gear the angle is in to obtain the knob gear. In the example shown in Figure 4, the angle is $139^{\circ }$, then the gear is right top, and the angle is $94^{\circ }$, then the gear is top. Section 3.1 introduces the knob area positioning algorithm based on YOLOv4 in detail. Section 3.2 presents the Darknet53-DUC-DSNT detection algorithm of knob key points in detail. Section 3.3 introduces the classification method of knob gears.

### 3.1. The Knob Area Positioning Based on YOLOv4

In this article, YOLOv4 [15,18] is used as the area positioning network to locate and segment the knob area. Compared with other models, although Mask-RCNN [19] and Cascade R-CNN [20] have high detection accuracy but slow speed, small models are fast but not accurate. Some models require many GPUs for parallel training because the networks are too large, which requires high hardware. YOLOv4 is a real-time and high-precision target detection model and can be trained on only one GPU. YOLOv4 is used to detect knobs on our original collected images, and the recognition accuracy is at least 98%, which fully meets the needs of target detection in this article.

The YOLOv4 target detector is divided into the backbone responsible for extracting features, the neck responsible for transmission to the target detection part, and the head responsible for target detection. YOLOv4 uses the feature extraction network CSPDarknet53 that references CSPNets (cross-stage partial networks) [39] based on Darknet53. It first divides the feature map of the base layer into two parts and then merges them through a cross-stage hierarchy. It guarantees accuracy and solves the problem of repeated gradient information for network optimization in other large convolutional neural networks.

The role of the neck is to enrich the information input to the head through adding or concatenating bottom-up and top-down adjacent feature maps by element. Therefore, the input of the head contains rich bottom-up spatial information and top-down semantic information. The neck mainly uses the SPP-Net (spatial pyramid pooling network) [40] structure to solve how feature maps of different sizes enter the fully connected layer. The max-pooling kernel size of SPP-Net is k = {1×1,5×5,9×9,13×13}, and the pooled feature maps from different kernel sizes are concatenated together as output. SPP layers can increase the receptive field of the backbone network more effectively than max pooling with single kernel size and then fuse feature map through PANet (path aggregation network) [41] structure. After that, the neck passes the image features to the prediction layer to predict the image features.

**Figure 5:**
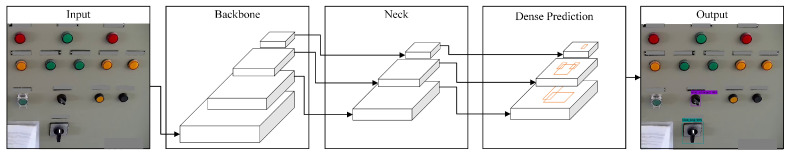
YOLOv4 knob area positioning algorithm.

As shown in Figure 5, the CSPDarknet53 backbone network extracts the features of the original images collected from the substation. Then, the features are transferred to the neck. The neck uses SPP-Net and PANet to generate a hierarchical structure of feature maps with different spatial resolutions to detect objects of different scales, increase the receptive field and perform feature map fusion, and enrich spatial and semantic information. Finally, this information is sent to the head responsible for object detection for prediction, generating bounding boxes and predicting categories, and locating the knob area.

### 3.2. The Knob Key Point Detection Based on Darknet53-DUC-DSNT

**Figure 6:**
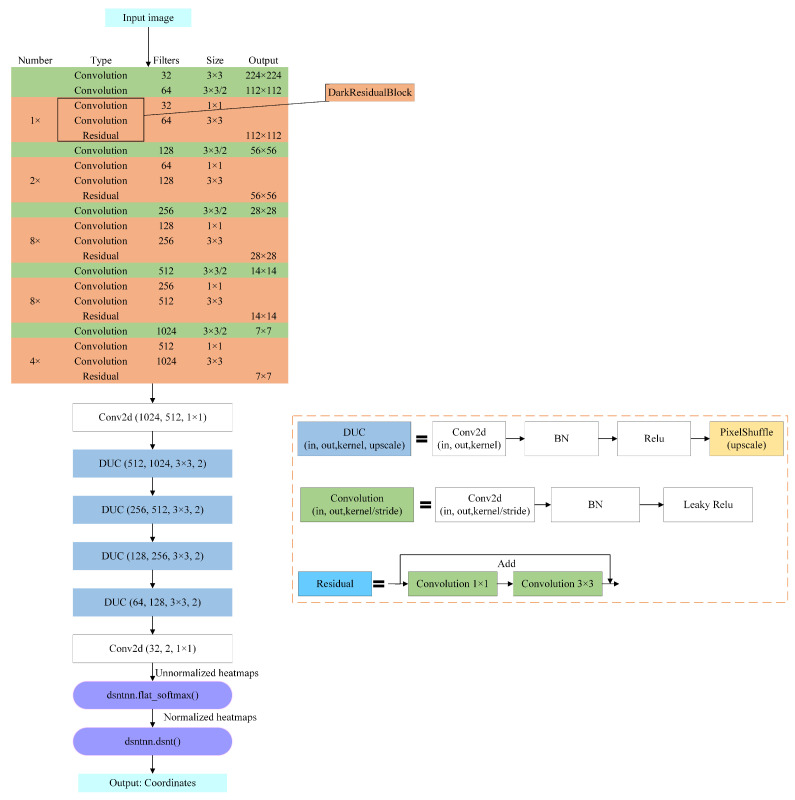
Darknet53-DUC-DSNT model structure for knob key point detection. The input image is extracted through Darknet53, upsampled through 4 DUCs, and finally returns key point coordinates through DSNT.

The Darknet53-DUC-DSNT model structure diagram for knob key point detection is shown in Figure 6. The three-channel image is input into Darknet53 for feature extraction, and then the detailed information is recovered through the DUC structure. Finally, the spatial generalization ability is enhanced through the DSNT structure to obtain the key point coordinates. The exact process is as follows.

Firstly, we use Darknet53 [18] as the backbone network for keypoint detection, which has higher training accuracy than Darknet19 [42,43,44] and higher efficiency than ResNet101 [45,46] and ResNet152 [47,48,49,50] networks. Combining the characteristics of ResNet [51], Darknet53 avoids the gradient problem caused by the deep network while ensuring the strong expression of features. Therefore, considering the accuracy and efficiency, we chose Darknet53 as the backbone network. In the article [18], the input picture size recommended by the author is 416×416, but according to the pictures collected in the actual scene, we set the input picture size to 224×224. After the first convolution of Darknet53, the feature map size does not change, but the number of channels becomes 32. A large number of DarkResidualBlocks are stacked in the following Darknet53 network. There are five groups of repeated DarkResidualBlock structures in total, and the repetition times are 1, 2, 8, 8, and 4, respectively. A convolution with a stride size of 2 and a kernel size of 3×3 is inserted between every two DarkResidualBlocks to complete the downsampling operation. The entire backbone network is reduced in dimension 32 times, and the final output features map dimension is 7. DarkResidualBlock uses a lot of 1×1 convolution and 3×3 convolution for channel expansion or reduction. Residual uses 1×1 convolution to shrink the channel and then uses 3×3 convolution to restore the channel, whose essence is matrix decomposition for reducing the number of parameters.

Then, since there are many images with low SNR and the images will lose many details due to downsampling after Darknet53, we introduced the DUC structure to restore the lost information. After the feature map passes through the last DarkResidualBlock structure of Darknet53, the output channel becomes 1024, which is necessary to compress the number of channels to 512 through a 1×1 convolution and then input to the DUC structure. This model uses 4 DUCs, upsampling the feature map four times through PixleShuffle in the DUC; each upsampling factor (upscale) is 2, converting the low-resolution feature map to a high-resolution feature map through convolution and multi-channel recombination and recovering detailed information.

Finally, it is input into the DSNT structure for coordinate regression prediction. After the feature map passes through the DUC structure, the Gaussian heatmap of each channel is obtained through a 1×1 convolution. Then the Gaussian heatmap of each channel is normalized so that the normalized Gaussian heatmap has only one peak and finally this peak is converted to obtain the coordinates of the key point. The advantage of the DSNT module is that it can predict the low-resolution Gaussian heatmap and make the gradient flow from the coordinate points to the Gaussian heatmap without adding extra computation. DSNT learns heatmaps indirectly by optimizing the loss of predicted coordinates output by the entire model, thereby enhancing spatial generalization.

To sum up, the general idea of the model is to extract the features of the knob image through Darknet53 first. After the image passes through Darknet53, we found that many details were lost, resulting in low recognition accuracy, so we used the DUC structure for upsampling to restore the details. Although the detailed information of the feature map was restored, the spatial generalization ability was weak, resulting in recognition accuracy still not being high, so the DSNT structure was introduced to enhance the spatial generalization ability. The final Darknet53-DUC-DSNT detection model of knob key points has good performance and high recognition accuracy and controls the regression error of key points within 1 pixel.

#### 3.2.1. DUC

**Figure 7:**
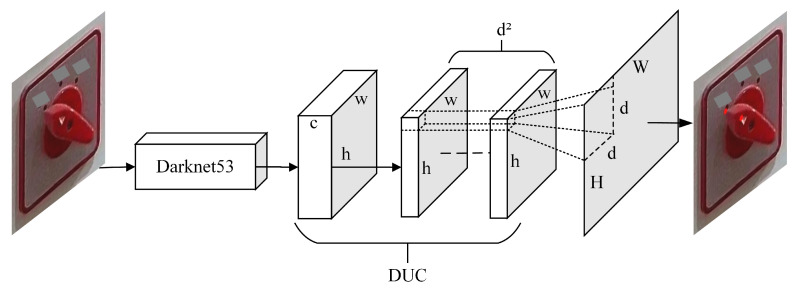
DUC structure.

The article [23] proposed dense upsampling convolution, a method of manipulating convolutional correlation operations. The specific structure of the DUC is shown in Figure 7. Assuming that the height of the original image is *H*, the width is *W*, and the color channel is *C*, the dimension becomes h×w×c after Darknet53, where h=H/d and w=W/d, and *d* is called the downsampling factor. The DUC operation is based on convolution; after convolution, the dimension of the output feature map is $h\times w\times (d^{2}\times L)$, and then the size of H×W×L is obtained by reshaping, where *L* is the number of categories of key points. It can be seen that the idea of DUC is to compensate for the loss in the length and width dimensions through the channel dimension. DUC divides the entire label map into sub-parts of the same size as the input feature map. All the sub-parts are superimposed $d^{2}$ times to generate the entire label map. For convolutional neural networks, the semantic information of large objects appears in the deeper feature map, and the semantic information of small objects appears in the shallower feature map. Since the Darknet53 network is relatively deep, a large part of the detailed information will be lost, which is very unfavorable for the detection of fine-grained features of images. The DUC amplifies the downsampled feature map to the desired size by learning some amplified filters. Each of its dense convolutions is learning the prediction of each pixel, thereby recovering the details of the image.

#### 3.2.2. DSNT

The key point detection of the knob is essentially a numerical coordinate regression task, which returns the coordinates of two key points of a knob. At present, there are two methods for mainstream regression of key points.

1. The fully connected layer is used to return the coordinate points directly. The advantage of this approach is that the training speed is breakneck, and it is an end-to-end full differential training. The disadvantage is that the spatial generalization ability is weak, the weight obtained by the full connection method depends heavily on the distribution of training data, which is very easy to cause overfitting, and the accuracy is not high in the case of high resolution.
2. The predictive Gaussian heatmap method. The advantage of this method is that the accuracy is usually higher than that of method 1. The disadvantage is that the method is not a fully differential model from input to output, and the accuracy is lower in low resolution.

The different feature distributions of different kinds of knobs and the existence of many oblique views interfere with feature extraction and reduce the spatial generalization ability. Therefore, inspired by the article [26], the DSNT module used in this article can have full differential training and better spatial generalization capabilities.

Suppose that the input of DSNT is (batchSize,H,W,3), and the output is (batchSize,H/2,W/2,2), representing the regression of 2 key points, represented by *Z*. DSNT acts on each channel, and the output is (batchSize,2,2), representing the two key points’ *x* and *y* coordinates. The Gaussian heatmap output by each channel is normalized, defined as $\hat{Z}$, where $\hat{Z}$ is an m×n matrix, and the expression is (1). The activation function used for normalization is the softmax; the formula is (2). The purpose of normalization is to make the input of DSNT a discrete probability distribution.

(1) $$\hat{Z}=\phi \left(Z\right)$$

(2) $$Z_{i,j}^{\prime }=exp\left(Z_{i,j}\right)$$

Define two m×n matrices *X* and *Y*, where m=W, n=H, i=1…m, j=1…n.

(3) $$X_{i,j}=\frac{2j-(n+1)}{n}$$

(4) $$Y_{i,j}=\frac{2i-(m+1)}{m}$$

(5) $$x={\langle \hat{Z},X\rangle }_{F}$$

(6) $$y={\langle \hat{Z},Y\rangle }_{F}$$

Equations (3) and (4) can redistribute the coordinate values of the *X* and *Y* matrices to (−1, 1). If the normalized Gaussian heatmap has only one peak, then the transformation methods of (5) and (6) can directly obtain the *x* and *y* values. Since $\hat{Z}$ is normalized, the probability distribution condition is satisfied and can obtain the joint probability distribution Formula (7) of random variables *X* and *Y*.

(7) $$\mathrm{Pr}\left(c=\left[X_{i,j}\;Y_{i,j}\right]\right)={\hat{Z}}_{i,j}$$

where *c* is the output coordinate of a channel, and the coordinate value obtained after DSNT transformation is the mean value of the joint distribution of Formula (7), as shown in Formula (8).

(8) μ=E[c]

Combined with the above formula, the output value of the DSNT module can be obtained, as shown in Formula (9).

(9) $$DSNT\left(\hat{Z}\right)=\mu =\left[{\langle \hat{Z},X\rangle }_{F}\;{\langle \hat{Z},Y\rangle }_{F}\right]$$

**Figure 8:**
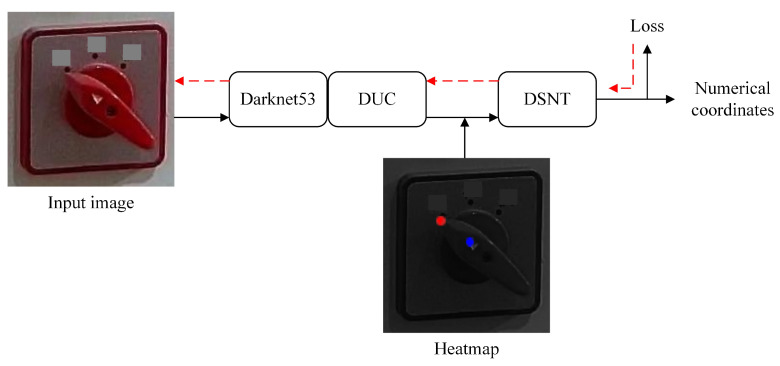
DSNT structure.

The DSNT structure diagram is shown in Figure 8.

### 3.3. Knob Gear Classification

Our knob gear recognition model is a three-stage model, including knob positioning, key point detection, and gear classification. After positioning and detection, we obtain the coordinates of two key points of the knob: the rotating center point and the pointing point. Gear classification is divided into two steps: calculate the angle and then judge the gear.

The first step is constructing a straight line between two key points and calculating the angle between the straight line and the horizontal direction. We use Formulas (10) and (11) to calculate the angle; $\left(x_{1},y_{1}\right)$ are the coordinates of the center point of the knob, and $\left(x_{2},y_{2}\right)$ are the coordinates of the pointing point of the knob. The atan2 function returns the azimuth angle from the origin to the point $\left(y_{2}-y_{1},x_{2}-x_{1}\right)$, that is, the angle with the x-axis, in radians, and the value range is [−π,π]. Then we convert radians to degrees.

(10) $$\theta =atan2\left(y_{2}-y_{1},x_{2}-x_{1}\right)\times \frac{180}{\pi }$$

(11) $$atan2\left(y,x\right)=\left\{\begin{array}{ccc} \arctan(\frac{y}{x}) & \; & x>0\\ \arctan(\frac{y}{x})+\pi & \; & y\geq 0,x<0\\ \arctan(\frac{y}{x})-\pi & \; & y<0,x<0\\ \frac{\pi }{2} & \; & y>0,x=0\\ -\frac{\pi }{2} & \; & y<0,x=0\\ undefined & \; & y=0,x=0 \end{array}\right.$$

The second step is to determine which gear the angle falls in and find the gear of the knob. According to the actual situation, the judgment of the gear is allowed to have an error of $\pm 5^{\circ }$. The gear and angle range are shown in Table 1.

## 4. Experiment

### 4.1. Experimental Environment

The experimental platform of this article is the Ubuntu 20.04.3 LTS operating system, and the equipment used for model training is two NVIDIA GeForce RTX 2080, both of which have 8G video memory and CUDA 11.4. The programming language is Python 3.6, and the deep learning framework is Pytorch 1.2.

### 4.2. Experimental Dataset and Data Augmentation

This experimental dataset comes from the daily inspection collection of the Guyang Xingshun West Wind Farm substation. This dataset belongs to a dataset in a specific scenario of engineering applications involving the relevant intellectual property rights of the company and is a non-public dataset. In the knob area positioning network based on YOLOv4, the dataset has 1025 cabinet panel pictures. We identified the knob area from 1025 pictures and segmented them to obtain a total of 1480 knob pictures. We randomly sampled knob datasets at a ratio of 7:1:1:1 for rotation, additive Gaussian noise, filtering, and sharpening, and can obtain a total of 20,766 images after data augmentation. We randomly selected the knob dataset according to the ratio of 8:1:1 to form the training set, validation set, and test set. The knob dataset in the detection network of knob key points based on Darknet53-DUC-DSNT is shown in Table 2.

To ensure the model’s generalization and balance the judgment of various knobs, the initial knob dataset needs to be augmented. Since the knob gears collected from the substation only have two to three gears, the number of gears is minimal. The dataset is randomly rotated from $-180^{\circ }$ to $180^{\circ }$ to balance the number of pictures of each gear of the knob and generate eight gears: top, bottom, left, right, left top, left bottom, right top, and right bottom. Random additive Gaussian noise, Gaussian filtering, and sharpening processing are applied to the dataset to balance image quality and reduce the impact of lighting conditions. The knob dataset after data augmentation has a total of 20,766 pictures, and the distribution of various types of knobs before and after augmentation is shown in Table 3.

### 4.3. Performance Evaluation Criteria

Aiming at the detection model of knob key points based on Darknet53-DUC-DSNT, we formulate the performance evaluation index based on the root mean square error (RMSE) and the error range that can be tolerated in practical engineering. RMSE is used to measure the deviation between the test value and the actual value, and its formula is shown in Formula (12). $h(x_{i})$ is the predicted value, $y_{i}$ is the actual value, and *m* is the number of key points.

(12) $$RMSE\left(X,h\right)=\sqrt{\frac{1}{m}\sum_{i=1}^{m}{\left(h\left(x_{i}\right)-y_{i}\right)}^{2}}$$

We test on the test set of knobs, combined with the acceptable error range of the actual engineering, limiting the error range between 0 and 1.0. If the RMSE value obtained by the test is greater than 1.0, we consider the test result to be outside the acceptable error range, and the picture is a negative sample. If the RMSE value obtained by the test is less than or equal to 1.0, we consider the test result to be within the acceptable error range, and the picture is a positive sample. Finally, we calculate the average RMSE of all the pictures in the knob test set, and combine the proportion of positive samples to the total samples to represent the accuracy of comprehensively judging the model’s performance. The smaller the average RMSE, the smaller the error between the predicted and actual values. The larger the number of positive samples, the higher the proportion of accurate prediction results.

For the classification of knob gears, we use three indicators of recall, precision, and F-score to evaluate the classification results of several models. The true positive (TP), false negative (FN), and false positive (FP) are used to calculate the recall, precision, and F-score. Recall indicates the proportion of the predicted positive samples that are actually positive samples, as shown in Equation (13). Precision represents the proportion of positive samples that are expected to be correct, and the calculation formula is (14). F-score is the weighted harmonic average of precision and recall. In our article, F1 measures precision and recall comprehensively. The calculation formula of F1 is (15).

(13) $$Recall=\frac{TP}{TP+FN}\times 100\%$$

(14) $$Precision=\frac{TP}{TP+FP}\times 100\%$$

(15) $$F1=\frac{2\times Recall\times Precision}{Recall+Precision}\times 100\%$$

### 4.4. YOLOv4 Knob Area Positioning Results

**Figure 9:**
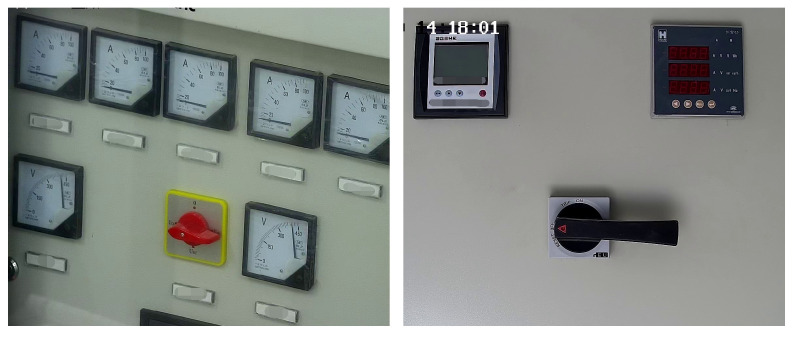
Original cabinet panel images collected on substation.

**Figure 10:**
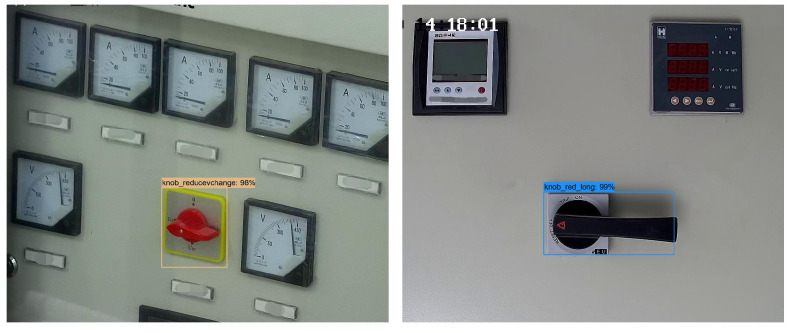
YOLOv4 knob areas positioning results.

**Figure 11:**
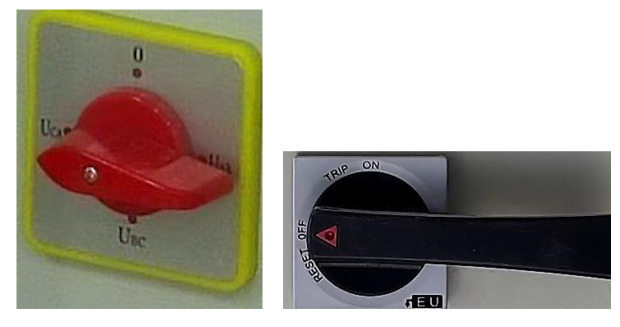
Cropped knob area images.

We used the labeling tool to mark the knob area of the cabinet panel to generate the corresponding XML file. Then, the YOLOv4 trained the cabinet panel dataset and generated the model file after completing the training. We input the test picture, and it predicted and returned the coordinates and category of each knob. Figure 9 has the original cabinet panel images. Figure 10 shows the results of YOLOv4 area positioning, and we can see that the recognition accuracy of the two knobs reaches 98% and 99%, respectively, which fully meets our needs. Figure 11 has two cropped knob area images.

### 4.5. Darknet53-DUC-DSNT Knob Key Point Detection Results and Comparison

In order to verify the accuracy of the detection model of knob key points based on Darknet53-DUC-DSNT, with the DUC and DSNT both being essential components, we conducted ablation experiments, comparing the Darknet53-DUC-DSNT model with the Darknet53 model, Darknet53-DUC model, and Darknet53-DSNT model. The model comparison results are in Table 4.

We input the test image to the detection model of the knob key point, and it predicted and returned the coordinates of each knob. The green pixels are the marked points, and the red pixels are the predicted points. The experimental results can be concluded as follows:

1. The knob key point model proposed in our article has the best prediction effect, the predicted points almost coincide with the marked points, and the average RMSE of all knobs is less than 1. All types of knobs achieved good detection results, whether front or oblique.
2. Although the accuracy rates of the Darknet53, Darknet53-DUC, and Darknet53-DSNT models are very low, the average RMSE is not high, indicating that the average prediction error of each picture is not high. Still, the error exceeds 1 pixel, leading to low accuracy.
3. Compared with the Darknet53 model, Darknet53-DUC improves the detection accuracy by 0.301 pixels on average. For each kind of knob, the average RMSE of the Darknet53-DUC model is smaller than the average RMSE predicted by the Darknet53 model, indicating that DUC effectively enhances the feature extraction ability, which improves the detection accuracy.
4. Compared with the Darknet53 model, the average RMSE of Darknet53-DSNT is higher, indicating that using DSNT under the premise of losing detailed features of the image will cause the opposite effect. Hence, DUC is essential, and we must first restore the image feature information.
5. The average RMSE of the Darknet53-DUC-DSNT model is 0.549 pixels lower than that of Darknet53-DUC, indicating that the use of DSNT in the case of image detail feature recovery can effectively enhance the spatial generalization ability, so DSNT is also essential.

### 4.6. Knob Gear Classification Results and Comparison

The Darknet53-DUC-DSNT model, Darknet53 model, Darknet53-DUC model, and Darknet53-DSNT model all performed for 300 iterations and set the learning rate to 0.001. In the model of method [21], due to the small MobileNet, to prevent overfitting, we only performed 100 iterations and set the learning rate to 0.001.

**Figure 12:**
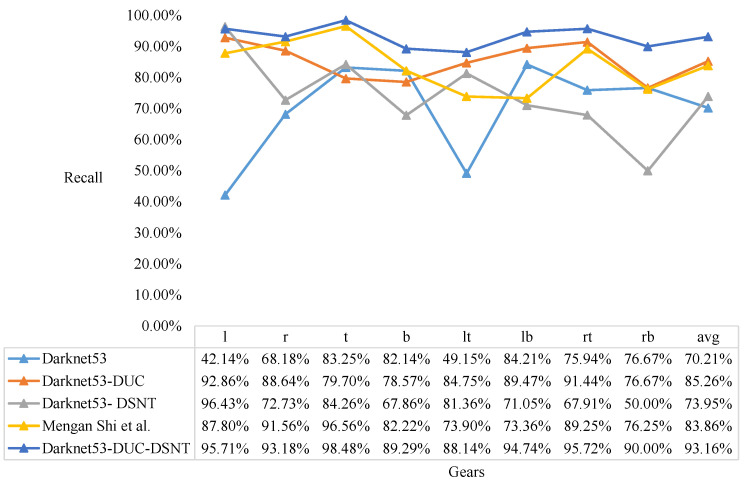
Recall results of knob gear classification. Comparison of Darknet53, Darknet53-DUC, Darknet53-DSNT, Mengan Shi [21], and our model.

**Figure 13:**
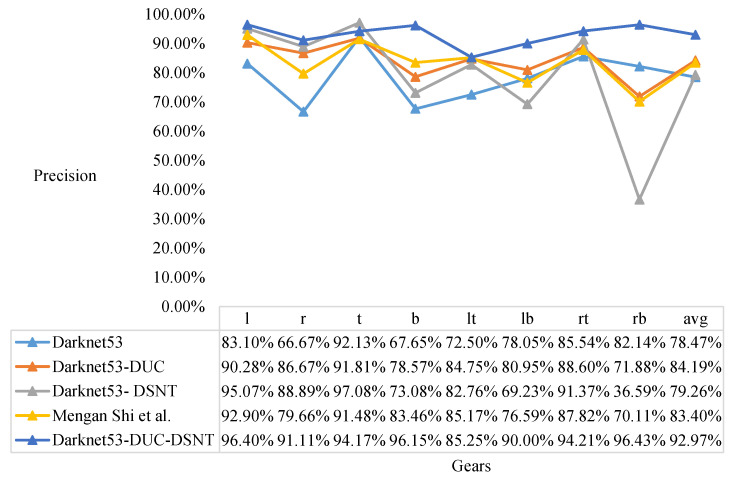
Precision results of knob gear classification. Comparison of Darknet53, Darknet53-DUC, Darknet53-DSNT, Mengan Shi [21], and our model.

**Figure 14:**
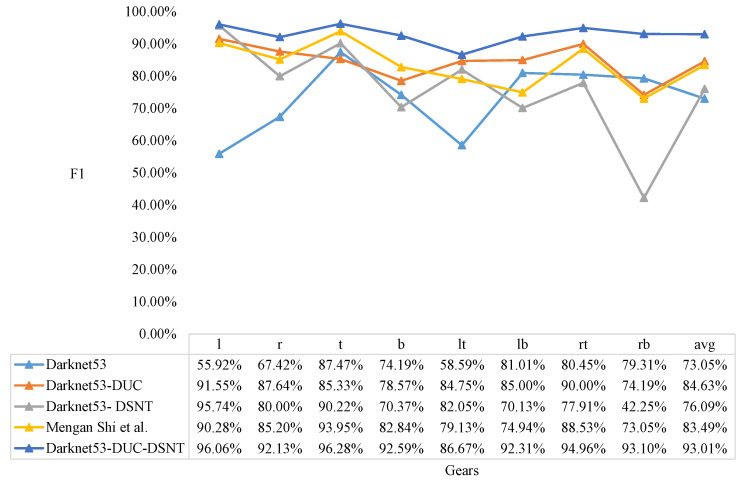
F1 results of knob gear classification. Comparison of Darknet53, Darknet53-DUC, Darknet53-DSNT, Mengan Shi [21], and our model.

We compare the Darknet53, Darknet53-DUC, Darknet53-DSNT, Mengan Shi [21], and our model and test the results of five models for classification. The results of the knob gear classification are shown in Figure 12, Figure 13 and Figure 14.

It can be seen from Figure 12 that the model proposed in this paper has the highest recall in each gear, and the average recall is also the highest. It can be seen from Figure 13 that the model proposed in this paper has a slight disadvantage in the precision of t (top gear) compared with the Darknet53-DSNT model, but the precision in other gears is the best, and the average precision is also the best. As can be seen from Figure 14, the comprehensive results of the recall and precision, the prediction results of the model proposed in this paper are the best in each gear, and the average F1 is also the highest.

From the results of F1, it can be concluded that the Darknet53-DSNT model improves the performance of the Darknet53 model by 3.04%, and the Darknet53-DUC model improves the performance of the Darknet53 model by 11.58%. We combine DUC and DSNT to enhance the performance of the Darknet53 model by 19.97%, and the performance of our three-stage model is 9.52% higher than that of the two-stage model proposed in the paper [21].

## 5. Conclusions

We are aiming at the problems of missing image features, interference in feature extraction, weak spatial generalization ability of the model, false knob gear detection, and incompatibility of the algorithm. We combine the YOLOv4 target detection algorithm with the improved Darknet53 key point detection algorithm to form a knob gear detection model. The YOLOv4 is used to detect the knob area of the cabinet panel image collected from the substation and crop the knob area. We proposed the Darknet53-DUC-DSNT model to detect the key points of the knob image, regress the coordinates of two key points, and calculate the angle to obtain the knob gears. Our model dramatically improves detection performance while ensuring real-time performance. We also propose a model evaluation index based on the RMSE and acceptable error range of the actual engineering of the substation. In the subsequent work, we will focus on reducing the model size and improving the detection speed. Therefore, it is necessary to conduct further research on substation data and integrate more image recognition methods into practical application scenarios to improve the algorithm’s generality and the model’s performance.

## Figures and Tables

**Table 1:** Gear and angle range.

Direction	Angle Range/${}^{{}^{\circ}}$
Top	(85, 95)
Bottom	(−95, −85)
Left	(−5, 5)
Right	(−180, −175) or (175, 180)
Left Top	(40, 50)
Left Bottom	(−50, −40)
Right Top	(130, 140)
Right Bottom	(−140, −130)

**Table 2:** Knob experiment dataset.

Dataset	Quantity
Initial knob total dataset	1480
Augmented knob total dataset	20,766
Augmented knob training set	16,618
Augmented knob verification set	2079
Augmented knob test set	2069

**Table 3:** Dataset distribution before and after knob augmentation.

Name	Picture	Before	After	Name	Picture	Before	After
Knob1	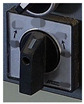	479	2146	Knob6	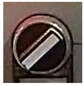	116	2070
Knob2	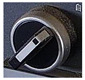	539	2152	Knob7	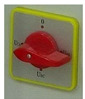	24	2040
Knob3	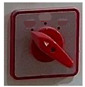	140	2070	Knob8	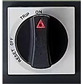	24	2040
Knob4	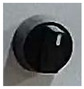	66	2080	Knob9	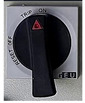	29	2025
Knob5	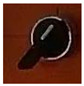	60	2100	Knob10	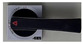	3	2043

**Table 4:** Comparison results of key point detection models.

	Model	Darknet53	Darknet53-DUC	Darknet53-DSNT	Darknet53-DUC-DSNT
Knob					
Knob1	Accuracy	17.76%	22.90%	12.62%	80.84%
	Avg RMSE	2.190	1.862	12.847	0.717
	Result	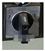	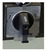	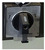	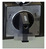
Knob2	Accuracy	63.80%	76.47%	63.80%	98.19%
	Avg RMSE	1.059	0.810	2.260	0.394
	Result	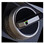	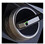	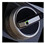	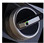
Knob3	Accuracy	44.77%	42.26%	97.91%	98.74%
	Avg RMSE	1.572	1.171	0.414	0.413
	Result	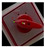	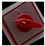	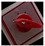	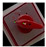
Knob4	Accuracy	84.44%	100.00%	100.00%	100.00%
	Avg RMSE	0.700	0.416	0.162	0.154
	Result	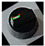	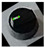	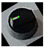	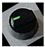
Knob5	Accuracy	100.00%	99.03%	90.82%	100.00%
	Avg RMSE	0.371	0.339	0.404	0.156
	Result	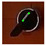	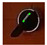	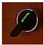	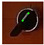
Knob6	Accuracy	81.69%	93.90%	51.64%	100.00%
	Avg RMSE	1.054	0.547	1.146	0.200
	Result	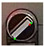	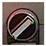	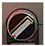	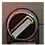
Knob7	Accuracy	46.60%	70.68%	73.30%	100.00%
	Avg RMSE	1.103	0.762	1.352	0.320
	Result	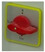	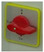	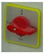	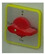
Knob8	Accuracy	66.67%	78.51%	71.49%	100.00%
	Avg RMSE	0.924	0.775	1.395	0.292
	Result	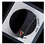	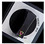	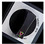	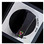
Knob9	Accuracy	36.36%	55.08%	64.17%	99.47%
	Avg RMSE	1.271	0.997	2.990	0.382
	Result	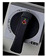	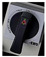	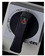	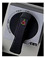
Knob10	Accuracy	8.99%	46.03%	67.20%	100.00%
	Avg RMSE	1.515	1.067	4.992	0.274
	Result	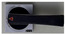	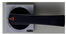	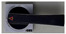	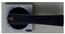
All knobs	Accuracy	54.20%	66.63%	67.79%	96.35%
	Avg RMSE	1.185	0.884	2.795	0.335
